# Optogenetic vision restoration in the face of secondary and tertiary remodeling in the rd1 mouse retina

**DOI:** 10.1016/j.ymthe.2025.07.056

**Published:** 2025-08-05

**Authors:** Steven Hughes, Jessica Rodgers, Moritz Lindner, Stuart N. Peirson, Robert J. Lucas, Mark W. Hankins

**Affiliations:** 1 Nuffield Laboratory of Ophthalmology, Sleep and Circadian Neuroscience Institute, Nuffield Department of Clinical Neurosciences, University of Oxford, Oxford, UK; 2 Kavli Institute for Nanoscience Discovery, University of Oxford, Oxford, UK; 3 Faculty of Biology, Medicine and Health, University of Manchester, Manchester, UK; 4 Institute of Physiology and Pathophysiology, Department of Neurophysiology, Philipps-Universität Marburg, Marburg, Germany; 5 Department of Ophthalmology, Philipps-Universität Marburg, 35037 Marburg, Germany

**Keywords:** optogenetics, vision restoration, degeneration, remodeling, gene therapy, bipolar cell, retina, eye

## Abstract

Photoreceptor loss in retinal degeneration is followed by progressive remodeling of the surviving retina, which may present a barrier to vision restoration. To determine the impact of remodeling on the retina’s suitability for therapeutic interventions, we track changes in visual code produced by the optogenetic actuator ReaChR expressed in ON-bipolar cells of the rd1 mouse at 3, 5, 9, and 12 months. Anatomical analyses confirm that these ages encompass phase II (photoreceptor degeneration) to phase III (inner retinal thinning, dysmorphia, pigment epithelium infiltration) remodeling. Multi-electrode array recording from retinal ganglion cells reveals that ReaChR-driven responses to a range of visual stimuli are stable across this age range. Response amplitude, sensitivity, and reproducibility all increased between 3 and 5 months, remaining consistent thereafter. Receptive field sizes, contrast sensitivity, and temporal frequency tuning showed minor changes with age. The diversity of retinal ganglion cell coding was maintained, reflected by the diversity captured by unsupervised functional clustering, with 11 distinct visual channels retained across ages. Our data indicate that remodeling does not significantly impair, and at early stages may even enhance, the surviving retina’s ability to support visual restoration. Clinical intervention thus remains viable throughout remodeling, suggesting a wide window in disease progression for therapeutic benefit.

## Graphical abstract

**Figure:**
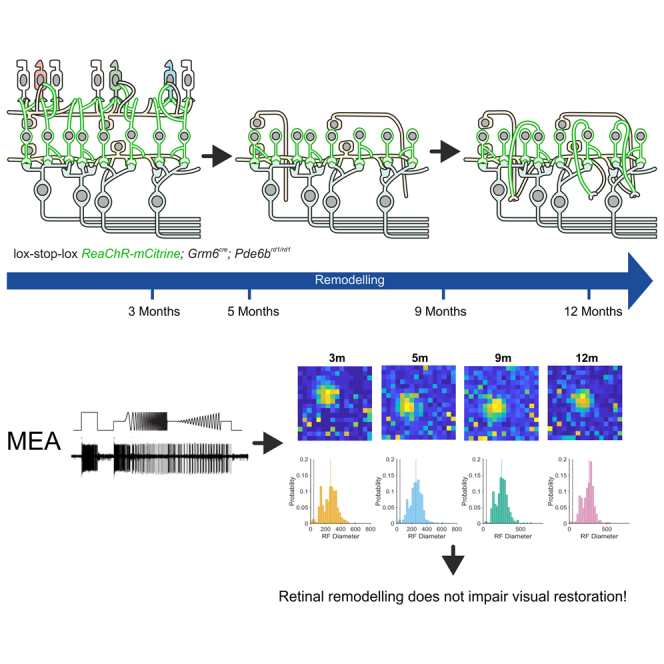

## Teaser

Optogenetic restoration of ON-bipolar cells supports robust and rich visual code across phase II to phase III retinal remodeling. Early remodeling enhances visual response sensitivity. The quality of restored vision is retained across later stages of remodeling. Optogenetic therapies targeting inner retina remain viable in the face of pronounced retinal remodeling.

## Introduction

Inherited retinal diseases (IRDs) are a common and still largely untreatable cause of vision loss.^1^^,^^2^^,^^3^ While the causes of IRDs are genetically diverse, the majority follow a common pattern of pathology, with an initial loss of rod photoreceptors followed by a secondary loss of cone photoreceptors leading to progressive and irreversible loss of vision. A common hallmark of IRDs is that initial cell loss is largely restricted to outer retinal photoreceptors, with bipolar cells and retinal ganglion cells (RGCs), which are involved in the processing and transmission of visual signals, and their associated interneurons (horizontal cells and amacrine cells) remaining largely intact, at least until the latter stages of retinal degeneration.^4^^,^^5^

Optogenetic therapies offer a potential treatment for end-stage retinal disease by conferring photosensitivity to surviving neurons of the degenerating retina (for review, see Lindner et al., Stefanov and Flannery, Parnami and Bhattacharyya, and Busskamp et al.^6^^,^^7^^,^^8^^,^^9^). Successful vision restoration has been reported in animal models, including rodents,^10^^,^^11^^,^^12^^,^^13^^,^^14^^,^^15^^,^^16^^,^^17^^,^^18^^,^^19^ dogs,^20^ and primates,^21^^,^^22^ and following delivery of a range of optogenetic actuators. A number of clinical trials are under way in humans (NCT0255673629 [Retrosense/Allergan/AbbVie], NCT0332633630 [GenSight Biologics], NCT0494577231 [Nanoscope Therapeutics], and NCT0427813132 [Bionic Sight]), with early results showing some level of visual restoration in a patient following adeno-associated virus delivery of ChrimsonR (a derivative of channelrhodopsin) to RGCs.^23^

One potential challenge to the efficacy of not only optogenetic therapies but also other gene- and cell-based therapies aimed at treating vision loss in a range of degenerative retina conditions^24^^,^^25^ is the process of progressive network remodeling that occurs following the loss of rod and cone photoreceptors, comprising anatomical and functional changes and ultimately widespread neuronal death. This pattern of remodeling has been shown in animal models,^4^^,^^5^^,^^26^^,^^27^^,^^28^ human postmortem tissue,^29^^,^^30^^,^^31^^,^^32^ and optical coherence tomography (OCT) tissue imaging in living patients^33^^,^^34^ and is best described in the rd1 mouse model of retinal degeneration (for review see Kalloniatis et al.^35^), with these changes categorized into three distinct (but likely overlapping) phases.^4^^,^^5^^,^^36^ Phase 1 remodeling is characterized by the loss of rod photoreceptors but also includes activation of Müller glial cells, sprouting or pruning of cellular processes, and the formation of new connections between rod bipolar cells and horizontal cells with cones. Changes in gene and protein expression also occur, with aberrant expression of ionotropic glutamate receptors causing a proportion of ON bipolar cells to show properties of OFF bipolar cells.^37^^,^^38^ Phase 2 is characterized by the loss of cone photoreceptors, with bipolar cells now becoming completely deafferented and sprouting of processes from amacrine cells and RGCs.^5^^,^^26^^,^^28^^,^^39^ Phase 3 occurs after rod and cone photoreceptor loss is complete and represents the most advanced stage of retinal remodeling. Neurite outgrowth is evident from all retinal cell types, there is abundant synapse formation, increased cellular migration and re-organization, and ultimately, increased retinal cell death is observed.^4^^,^^5^ Early phase 3 is characterized by progressive neurite rewiring and increased Müller cell hypertrophy. Mid-phase 3 remodeling involves cell migration (along Müller cell surfaces), increased cell death, and the formation of ectopic synaptic microneuromas. Late phase 3 involves continued remodeling and widespread death of all retinal cell types, a thinning of the inner plexiform layer, and invasion of retinal pigment epithelium (RPE) cells into the retina.^4^^,^^5^^,^^35^^,^^36^

Phases 1–3 of remodeling have the potential to impact the quality of restored visual information by altering the physiological function, network connections, and viability of cells expressing optogenetic tools and those transmitting and processing their output. This is a special concern for therapies aiming to take advantage of residual retinal processing capacity (e.g., following delivery of optogenetic tools to ON bipolar cells).^12^^,^^16^^,^^18^^,^^40^^,^^41^^,^^42^^,^^43^^,^^44^^,^^45^^,^^46^^,^^47^^,^^48^ Thus, retinal remodeling in later stages of retinal disease may corrupt or otherwise negatively impact the remaining retina circuits to such an extent as to place limits on the types and fidelity of visual responses that can be supported and that may ultimately limit clinical outcomes of treatment and the success of clinical trials.^49^^,^^50^^,^^51^

To investigate the impact of progressive retina remodeling on retinal circuits and the properties of optogenetic restored vision, here, we use a transgenic model, ReaChR grm6 rd/rd, where the red-activatable variant of channelrhodopsin (ReaChR)^13^^,^^52^ is expressed in ON bipolar cells of retinally degenerate rd1 mice.^11^ In this line, ReaChR is expressed from birth, yet in this study, all animals were housed under conditions with light levels several orders of magnitude below the threshold for ReaChR activation (see materials and methods), and thus optogenetic light responses are not activated in these mice prior to *ex vivo* examination of retina function. We have previously reported that these ReaChR grm6 rd/rd mice retain a rich visual code at 5 months of age when remodeling is ongoing and incomplete.^11^ Now, we use this model to fully characterize the properties of optogenetic retina light responses in separate cohorts of ReaChR grm6 rd/rd mice aged 3, 5, 9, and 12 months of age, time points corresponding to well-established periods of remodeling in the rd1 retina, with a total loss of rods and major but incomplete loss of cones (phase 1, 3 months), complete loss of cones (phase 2, 5 months), and late-stage degeneration, where significant retina remodeling occurs (early phase 3, 9 months; mid- to late phase 3, 12 months).^4^

## Results

### Progressive degeneration and remodeling in ReaChR grm6 rd/rd retinas

**Figure 1:**
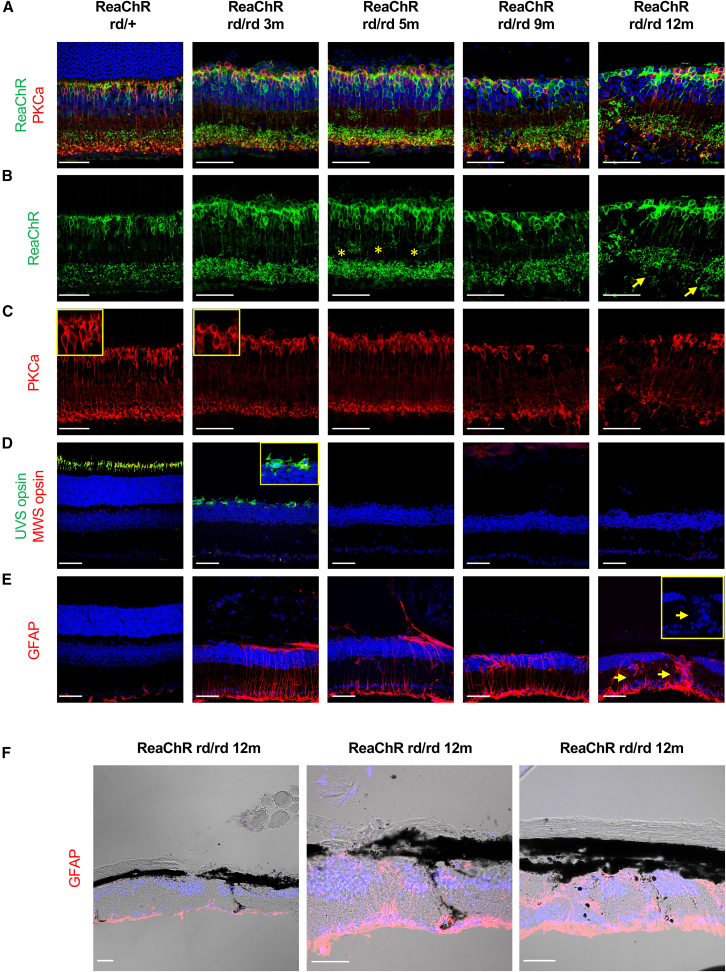
Anatomical changes and retina remodeling in ReaChR grm6 rd/rd retinas (A) Co-labeling for ReaChR.mCitrine (anti-GFP, green) and protein kinase C α (PKCα; red) in ReaChR grm6 rd/+ retina (non-degenerate) and ReaChR grm6 rd/rd retinas at 3, 5, 9, and 12 months, showing expression of ReaChR in rod and cone ON bipolar cells. (B) ReaChR (anti-GFP, green) staining showing the morphology of ReaChR expressing bipolar cells. The asterisks highlight increased sprouting of bipolar cell processes at 5 months. The arrows highlight areas of microneuroma formation. (C) PKCα (red) staining showing the morphology of rod ON bipolar cells. Highlighted boxes show loss of ON bipolar dendrites in ReaChR grm6 rd/rd retinas at 3 months compared to non-degenerate retina. (D) Co-labeling for UVS (green) and MWS (red) cone opsins showing cone loss in ReaChR grm6 rd/rd retinas at 3, 5, 9, and 12 months. Highlighted box shows surviving partially degenerate cones observed at 3 months. (E) GFAP (red) staining in ReaChR grm6 rd/rd retinas. Arrows and the inset show columns of cell nuclei (DAPI, blue) migrating along hypertrophic Müller cells at 12 months. (F) Merged fluorescence and transmitted light image showing GFAP (red) and DAPI (blue), highlighting the migration of pigmented RPE (appears black) into the inner retina in ReaChR grm6 rd/rd retinas at 12 months. Scale bars, 50 μm.

To confirm that our transgenic model followed the well characterized time course of degeneration in rd1 mice we collected retinae from ReaChR grm6 rd/rd at 3 months (postnatal day 90 [P90]), 5 months (P150), 9 months (P270), and 12 months (P360) of age for comparison with a matched cohort of visually intact animals (ReaChR grm6 rd/+). Across all ages, ReaChR.mCitrine was localized to cells positioned within the inner nuclear layer (INL) in ReaChR grm6 rd/rd and ReaChR grm6 rd/+ retinas, a subset of which also expressed the rod ON bipolar cell marker protein kinase C α (PKCα) (Figures 1A–1C) consistent with previous reports of ReaChR expression in rod and cone ON bipolar cells^11^ (see also Dhingra et al.^53^).

The outer nuclear layer (ONL) of ReaChR grm6 rd/rd retinas was largely absent by 3 months (Figure 1D), consistent with previous work with rd1 mice indicating complete rod and major cone loss by this age.^54^^,^^55^ Accordingly, immunohistochemistry (IHC) revealed a small number of surviving but partially degenerate cones (largely confined to the dorsal retina), staining for ultraviolet-sensitive (UVS) opsin but not medium-wavelength-sensitive (MWS) opsin, characteristically showing opsin expression mis-localized to cone cell bodies and loss of outer segments (Figure 1D). Progressive loss of the remaining cones was evident over remaining time points, with only a very small number of UVS-opsin-positive cones observed at 5 months and a virtually complete loss of cone staining observed at 9 and 12 months (Figure 1D).

Similarly, ReaChR grm6 rd/rd mice exhibit changes in bipolar cell morphology consistent with the known time course of retinal degeneration in the rd1 mouse model.^4^ By 3 months, PKCα staining indicated a widespread loss of rod ON bipolar cell dendrites in the remnant outer plexiform layer compared to non-degenerate retina (Figure 1C). Marc et al.^4^ reported morphological changes in bipolar cells in rd1 retina, and we also found evidence of sprouting of processes from ReaChR-positive ON bipolar cells consistent with the formation of new aberrant connections by 5 months (Figure 1B).^4^^,^^5^ Across 9 and 12 months, the morphology and localization of PKCα and ReaChR-positive cells in the ONL became increasingly disordered (although this progression was non-uniform across the retina), with these cells showing reduced density, more disorderly processes, and more variable localization within the INL (Figure 1B), alongside evidence of microneuroma formation (Figure 1B) consistent with progression to early, middle, and late phase 3-type remodeling events.^4^^,^^26^

To better visualize the transition to later stages of remodeling, we examined the expression of glial fibrillary acidic protein (GFAP) and followed structural changes in Müller cells that characterize the progression to late stages of retina remodeling.^4^^,^^26^^,^^27^^,^^56^^,^^57^^,^^58^ Similar to previous reports of rd1 mice^59^ ReaChR grm6 rd/rd retinas showed increased GFAP expression compared with non-degenerate retina (Figures 1E and 1F). At 3 months, ReaChR grm6 rd/rd retinas showed clear signs of reactive gliosis, with increased GFAP labeling restricted to Müller cell endfeet forming the inner limiting membrane, and Müller cell bodies with processes extending radially throughout the retina layers and forming an outer retina glial seal (Figures 1E and 1F).^59^ With increasing time, the disruption of retina cell layers and the migration of cells along columns of hypertrophic Müller cells became apparent in ReaChR grm6 rd/rd retinas at 9 months and more widespread at 12 months, where distinct areas of the retina show considerable disorganization (Figures 1E, 1F, and S1). Such retinal disorganization was the most apparent in areas of Müller cell invasion and extensive GFAP staining. The significant elevation of GFAP expression, disruption of retina layers, and mass migration of neurons along Müller cell columns, in combination with clear evidence of infiltration of RPE cells into the retina—evident at 12 months (Figures 1F and S1)—indicate a progression to mid- to late phase 3-type remodeling events.^4^^,^^26^

### Optogenetic light responses do not show age-dependent deterioration in rd/rd retinas

**Figure 2:**
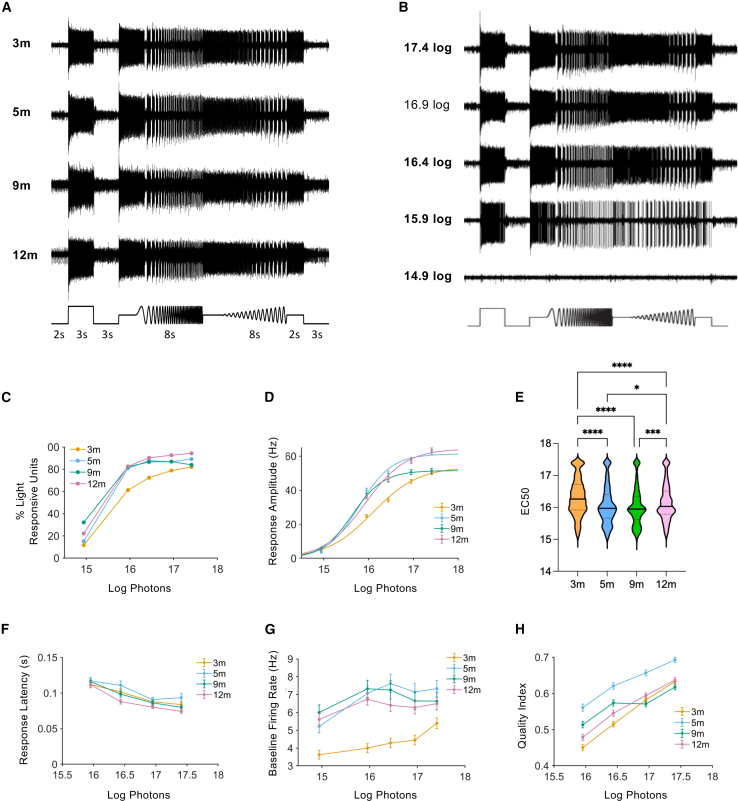
Properties of light responses in ReaChR grm6 rd/rd retinas (A) Examples of raw electrode data collected from ReaChR grm6 rd/rd retinas at 3, 5, 9, and 12 months following stimulation with the white light chirp stimulus (data shown for 16.9 log photons/cm^2^/s). (B) Example of raw electrode data showing ReaChR grm6 rd/rd responses to increasing intensity of light from 14.9 to 17.4 log photons/cm^2^/s (data shown for ReaChR grm6 rd/rd retinas at 5 months). (C) The percentage of total units classified as light responsive (LR) across light intensities (3 months, *n* = 1,082; 5 months, *n* = 774; 9 months, *n* = 797; 12 months, *n* = 883). (D) Graph showing mean response amplitudes across intensities (3 months, *n* = 1,082; 5 months, *n* = 774; 9 months, *n* = 797; 12 months, *n* = 883). (E) Response EC_50_ values of single LR units (3 months, *n* = 1,082; 5 months, *n* = 774; 9 months, *n* = 797; 12 months, *n* = 883). (F) Response latency across intensities (3 months, *n* = 976; 5 months, *n* = 711; 9 months, *n* = 785; 12 months, *n* = 876). (G) Baseline firing rate across intensities (3 months, *n* = 1,082; 5 months, *n* = 774; 9 months, *n* = 797; 12 months, *n* = 924). (H) Mean quality index (QI) values calculated for LR units at 3, 5, 9, and 12 months across intensities (3 months, *n* = 1,082; 5 months, *n* = 774; 9 months, *n* = 797; 12 months, *n* = 774). Graphs show mean ± SEM.

**Figure 3:**
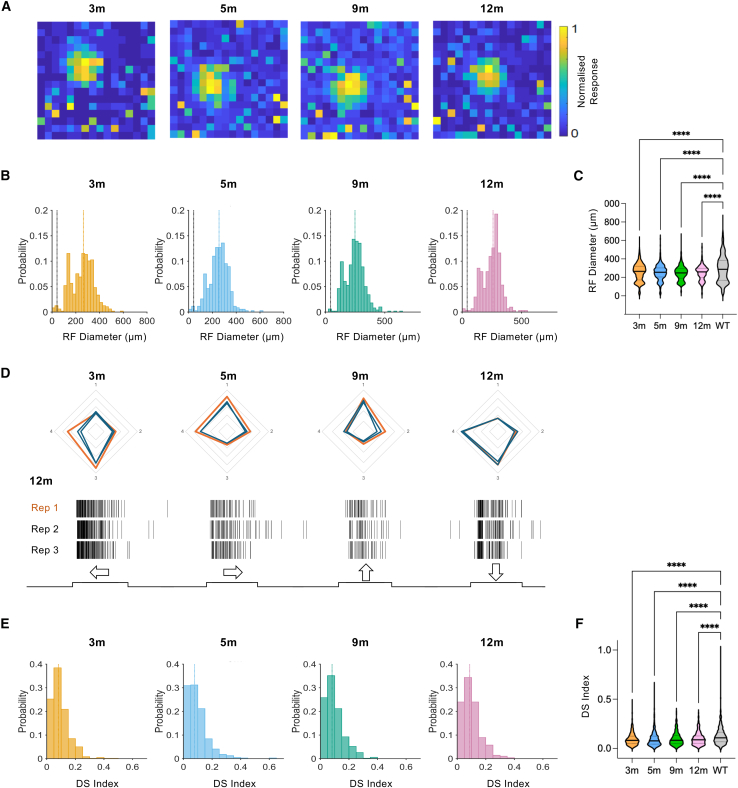
Responses to spatial stimuli in ReaChR grm6 rd/rd retinas (A) Example heatmaps showing normalized responses recorded at each location in a 16 × 16 grid to map receptive field (RF) diameters of single units from ReaChR grm6 rd/rd retinas at 3, 5, 9, and 12 months. (B) Histograms showing the distributions of RF values. Dashed line, median. Black dotted line, approximate size of spot from binary white noise stimulus. (C) Summary of RF diameters in ReaChR grm6 rd/d retinas, including comparison to non-degenerate WT retina (3 months, *n* = 477; 5 months, *n* = 346; 9 months, *n* = 495; 12 months, *n* = 301; WT, *n* = 586). (D) Polar plots showing examples of direction selective (DS) units recorded from ReaChR grm6 retinas at 3, 5, 9, and 12 months. Spike rasta plots are shown for the 12-month exemplar. Arrows show direction of moving bar stimuli. (E) Histograms showing the distributions of DS values. Dashed line, median. (F) Summary showing mean DS values calculated for individual LR units (3 months, *n* = 598; 5 months, *n* = 391; 9 months, *n* = 538; 12 months, *n* = 279; WT, *n* = 684).

We explored age-related changes in the visual code of ReaChR grm6 rd/rd retinas by using multi-electrode array (MEA) recordings to record changes in RGC spike firing (retina output) in a battery of analytical visual stimuli. These comprised a full-field white light chirp-type stimulus applied across a range of irradiances (see Figure 2),^60^ spatial sparse noise, and moving bars (see Figure 3).^11^^,^^41^ Robust, intensity-dependent responses to the chirp stimulus were observed from ReaChR grm6 rd/rd retinas at all ages (examples of raw data traces are shown in Figures 2A, 2B, and S2). While there is a clear effect of irradiance (two-way ANOVA, F(4, 12) = 149.0, *p* < 0.0001) and age (F(3, 12) = 10.35, *p* = 0.0012), there was no indication that the percentage of single units classified as light responsive (LR) decayed over age; they were actually slightly reduced at 3 months when compared to either 5, 9, or 12 months across the range of light intensities tested (Tukey’s post hoc test with multiple test correction, 3 vs. 5 months, *p* = 0.0160; 3 vs. 9 months, *p* = 0.0040; 3 vs. 9 months, *p* = 0.0013) (LR units at 16.9 log photons/cm^2^/s, 3 months = 78.8% [*n* = 1,039]; 5 months = 86.9% [*n* = 754]; 9 months = 86.9% [*n* = 825]; and 12 months = 92.6% [*n* = 907]) (Figure 2C).

As an initial examination of response properties, we analyzed responses to the light step portion of the chirp stimulus. The mean step response amplitude (change in spike firing rate corrected for baseline; 200-ms bins) was dependent on irradiance (two-way ANOVA, F(4, 17,660) = 1,182, *p* < 0.0001), age (F(3, 17,660) = 91.90, *p* < 0.0001), and an age-by-intensity interaction (F(12, 17,660) = 9.64, *p* < 0.0001). This, however, was not indicative of an age-dependent deterioration; rather, mean response amplitude was significantly increased at 5 and 12 compared to 3 months, and to a lesser extent, 9 months (Tukey’s post hoc test with multiple test correction, 3 vs. 5 months, *p* < 0.0001; 3 vs. 9 months, *p* < 0.0001; 3 vs. 12 months, *p* < 0.0001; 5 vs. 9 months, *p* < 0.0001; 9 vs. 12 months, *p* = 0.0001) (Figures 2D and S2). Linear regression analysis of data collected at saturating irradiance (16.9 log photons/cm^2^/s) showed a linear trend over time, indicating a consistent increase in mean response amplitude with age (*R*^*2*^ = 0.0059, slope = 0.0066, F(1, 3,534) = 20.80, *p* ≤ 0.0001). The mean response amplitude at 3 months was 45.3 ± 1.2 Hz (*n* = 1,082); 5 months, 57.6 ± 1.7 Hz (*n* = 774); 9 months, 50.6 ± 1.5 Hz (*n* = 797); and 12 months; 57.3 ± 1.5 Hz (*n* = 924).

To determine whether there were age-dependent changes in sensitivity, we extracted the half-maximal effective concentration (EC_50_) values of intensity response relationships for the light step at single-unit resolution (Figure 2E). This did reveal a significant effect of age (one-way ANOVA, F(3, 3,532) = 47.11, *p* < 0.0001). Again, however, post hoc tests indicate that this is primarily accounted for by lower sensitivity at the 3-month time point (mean EC_50_ at 3 months = 16.1 (*n* = 1,082); 5 months = 15.8 (*n* = 744); 9 months = 15.6 (*n* = 797); 12 months = 15.9 (*n* = 883) log photons/cm^2^/s) (Tukey’s post hoc test with multiple test correction, 3 vs. 5 months, *p* < 0.0001; 3 vs. 9 months, *p* < 0.0001; 3 vs. 12 months, *p* < 0.0001), with sensitivity also somewhat lower at 12 months (5 vs. 12 months, *p* = 0.0146; 9 vs. 12 months, *p* < 0.0001). Mean EC_50_ values showed a linear relationship, indicating a small but significant increase in response sensitivity over time (*R*^*2*^ = 0.0115, slope = −0.00059, F(1, 3,534) = 41.21, *p* ≤ 0.0001).

Across the range of intensities at which we recorded widespread responses (15.9–17.4 log photons/cm^2^/s), response latency was inversely related to stimulus intensity and showed a small but significant dependence on age (two-way ANOVA, F(3, 12,692) = 51.22, *p* < 0.0001 for irradiance; F = 9.204, *p* < 0.0001 for age). The effect of age was accounted for by reduced latency at 12 months (and to a lesser extent, 9 months) compared to earlier time points (Tukey’s post hoc test with multiple test correction 3 vs. 12 months, *p* = 0.0136; 5 vs. 9 months, *p* = 0.0276; 5 vs. 12 months, *p* < 0.0001) (Figure 2F). Mean latency values measured at saturating irradiance (16.9 log photons/cm^2^/s) showed a linear trend, indicating a small but significant decrease in response latency over time (*R*^*2*^ = 0.0014, slope = −3.111e−5, F(1, 3,346) = 4.546, *p* = 0.0331). The mean response latency at 16.9 log photons/cm^2^/s at 3 months = 0.088 ± 0.004 s (*n* = 976), 5 months = 0.091 ± 0.003 s (*n* = 711), 9 months = 0.086 ± 0.003 s (*n* = 785), and 12 months = 0.08 ± 0.002 s (*n* = 876).

Rates of baseline spike firing (recorded for 2 s immediately prior to the onset of the initial light step at each intensity) were higher at 5–12 months compared to 3 months of age (two-way ANOVA, F(3, 17,865) = 54.36, *p* < 0.0001) (Tukey’s post hoc test with multiple test correction, 3 vs. 5 months, *p* ≤ 0.0001; 3 vs. 9 months, *p* ≤ 0.0001; 3 vs. 12 months, *p* ≤ 0.0001) (Figure 2G), consistent with the known impacts of progressive degeneration on spontaneous retinal activity in rd1 mice.^61^^,^^62^ There was also a small effect of light intensity on baseline firing rate (two-way ANOVA, F(4, 17,865) = 8.622, *p* < 0.0001), indicating an effect of light history within our experiments, but no significant interaction of age by irradiance (F(12, 17,865) = 1.435, *p* = 0.1416). Rates of baseline firing measured at a saturating irradiance (16.9 log photons/cm^2^/s) showed a significant linear trend, indicating a significant increase across ages (*R*^*2*^ = 0.002401, slope = 0.005218, F(1, 3,575) = 8.606, *p* = 0.0034).

To provide an overall measure of response reproducibility for individual units, we calculated a quality index (QI) for firing rate profiles across the 10 repeats of the chirp stimulus at each irradiance (where 1 = identical response every trial).^60^ QI showed modest effects of both irradiance and age and a significant interaction between these terms (two-way ANOVA, F(3, 14,248) = 232.5, *p* < 0.0001 for irradiance; F = 88.35, *p* < 0.0001 for age, and F(9, 14,248) = 5.477, *p* < 0.0001 for age × irradiance) (Figure 2H). Overall, QI values across intensities were lower at 3 months than any later age and highest at 5 months (Tukey’s post hoc test, 3 vs. 5 months, *p* ≤ 0.0001; 3 vs. 9 months, *p* ≤ 0.0001; 3 vs. 12 months, *p* = 0.0024; 5 vs. 9 months, *p* ≤ 0.0001; 5 vs. 12 months, *p* ≤ 0.0001). QI values measured at a saturating irradiance (16.9 log photons/cm^2^/s) show no linear trend across ages (*p* = 0.094).

### Receptive field diameters and direction selectivity are stable over age in rd/rd retinas

Retinal degeneration and advance retinal remodeling have been reported to lead to retraction of bipolar cell dendritic fields and reduction in the size of RGC receptive fields (RFs).^63^ To address this issue, we mapped spatial RFs using a sparse binary noise stimulus consisting of a single spot of light (565 nm, 16.2 log photons/cm^2^/s, 1:1,000 contrast) in a random position on a 16 × 16 grid (Figure 3A).^11^^,^^41^ The mean RF diameter of light-responsive units probed in this manner were unchanged across age in ReaChR grm6 rd/rd retinas (Figures 3B and 3C) (one-way ANOVA, F(3, 1,615) = 0.7746, *p* = 0.5082) and showed no linear trend across ages (*p* = 0.1786) (mean RF diameter at 3 months = 254.3 ± 4.4 μm [*n* = 477]; 5 months = 251.7 ± 4.5 μm [*n* = 346]; 9 months = 246.4 ± 3.6 μm [*n* = 495]; 12 months = 248.3 ± 4.5 μm [*n* = 301]). However, RF diameters in ReaChR grm6 rd/rd retinas were significantly reduced at all ages compared to the intact retina (wild-type [WT] retina at 5 months, RF diameter = 290.2 ± 5.9 μm [*n* = 586]) (one-way ANOVA, F(4, 2,200) = 16.39, *p* < 0.0001; Tukey’s post hoc test with multiple test correction, 3 months vs. WT, *p* ≤ 0.0001; 5 months vs. WT, *p* ≤ 0.0001; 9 months vs. WT, *p* ≤ 0.0001; 12 months vs. WT, *p* ≤ 0.0001) (Figure 3C).

Responses to moving bar stimuli revealed units showing direction selectivity (DS) in ReaChR grm6 rd/rd retinas at all ages (Figure 3D), although units showing strong DS were relatively rare. To determine how DS was impacted by degeneration, we calculated a DS index for each single unit. DS index values showed no age-dependent change in the degenerate retina (one-way ANOVA, F(3, 1,802) = 0.4517, *p* = 0.716) (Figures 3E and 3F) nor linear trend across ages (*p* = 0.249), but were significantly reduced compared to the intact retina (one-way ANOVA, F(4, 2,485) = 19.47, *p* < 0.0001) (Tukey’s post hoc test with multiple test correction, 3 months vs. WT, *p* ≤ 0.0001; 5 months vs. WT, *p* ≤ 0.0001, 9 months vs. WT, p ≤ 0.0001, 12 months vs. WT, *p* ≤ 0.0001) (Figure 3F) (mean DS values at 3 months = 0.095 ± 0.003 [*n* = 598], 5 months = 0.095 ± 0.004 [*n* = 391], 9 months = 0.098 ± 0.003 [*n* = 538], 12 months = 0.099 ± 0.004 [*n* = 279], WT = 0.125 ± 0.003 [*n* = 684]).

### Functional diversity in response to light step

**Figure 4:**
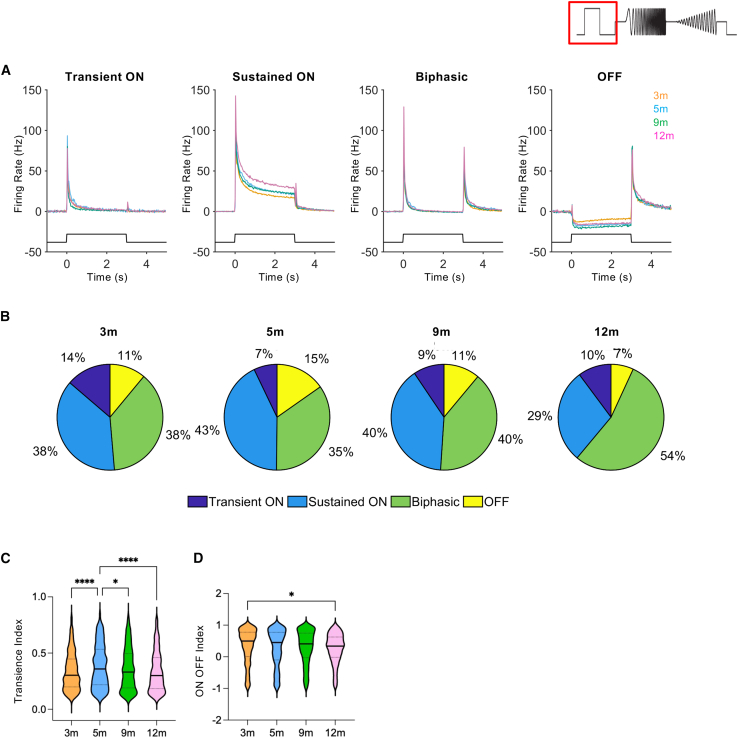
Diversity of light responses to a step stimulus in ReaChR grm6 rd/rd retinas (A) Mean PSTH traces for transient ON, sustained ON, biphasic, and OFF responses in ReaChR grm6 rd/rd retinas at 3, 5, 9, and 12 months. (B) Pie charts showing the distribution of response types across time points (3 months, *n* = 853; 5 months, *n* = 590; 9 months, *n* = 678; 12 months, *n* = 727). (C) Transience index values from individual LR units at 16.9 log photons/cm^2^/s (3 months, *n* = 1,039; 5 months, *n* = 754; 9 months, *n* = 825; 12 months, *n* = 907). (D) ON-OFF index values from individual LR units at 16.9 log photons/cm^2^/s (3 months, *n* = 1,039; 5 months, *n* = 754; 9 months, *n* = 825; 12 months, *n* = 907).

We next sought to examine how increasing retina remodeling might affect the diversity of light responses across age by exploring activity at a single irradiance (16.9 log photons/cm^2^/s) that drives robust responses at all ages. We have previously reported that RGCs in the ReaChR grm6 rd/rd retinas at 5 months show a diversity of responses to a light step encompassing ON sustained, ON transient, biphasic (ON/OFF), and OFF-type responses.^11^ We found that units could be classified according to these fundamental response types (see materials and methods) at all ages and that mean firing rate profiles for each category were qualitatively similar in ReaChR grm6 rd/rd retinas at 3, 5, 9, and 12 months (Figure 4A). Heatmaps showing the activity of individual units are shown in Figure S3. The overall proportion of light-responsive units classified as ON sustained, ON transient, biphasic, or OFF responses was comparable across ages, although an age-dependent effect was evident (chi-squared, df = 94.24, 9, *p* ≤ 0.0001) with a small increase in the percentage of biphasic units and a reduction in sustained ON units (and to a lesser extent, OFF units) evident at 12 months (Figure 4B).

The overall retention of response types across ages was apparent also following quantification of response transience (transience index, where a value of 1 is fully sustained) and response polarity (ON-OFF bias index, where a value of 1 is entirely ON) at single unit level (methods derived from Farrow and Masland^64^). Ranges for both these parameters were comparable across ages (Figures 4C and 4D), although the transience index was modestly increased at 5 months (one-way ANOVA, F(3, 3,521) = 11.80, *p* < 0.0001) (Tukey’s post hoc test, 3 vs. 5 months, *p* ≤ 0.0001; 5 vs. 9 months, *p* = 0.0259; 5 vs. 12 months, *p* ≤ 0.0001) (Figure 4C), but it showed no linear relationship across ages (*p* = 0.2598). ON-OFF bias values also varied between 3 and 12 months (one-way ANOVA, F(3, 3,521) = 3.285, *p* = 0.0199) (Tukey’s post hoc test, 3 vs. 12 months, *p* = 0.0139) (Figure 4D), but in this case showed a linear trend indicating a small but significant reduction in ON-biased responses over time (*R*^*2*^ = 0.0018, slope = −0.00021, F(1, 3,523) = 6.234, *p* = 0.013).

### Modest changes in temporal frequency tuning over age in ReaChR grm6 rd/rd retinas

**Figure 5:**
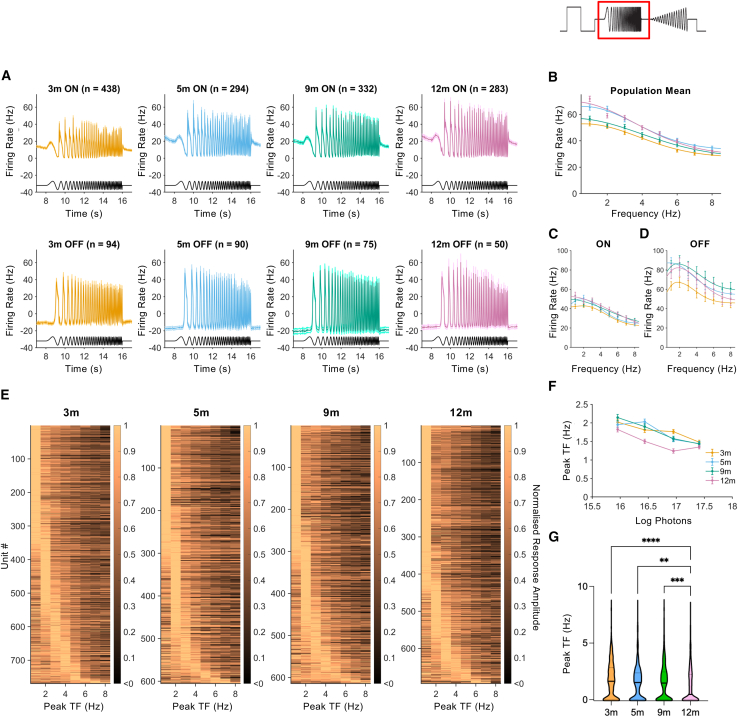
Temporal frequency tuning in ReaChR grm6 rd/rd retinas (A) Mean PSTH traces showing responses to modulations of temporal frequency from ON-responsive units (top) and OFF-responsive units (bottom) of ReaChR grm6 rd/rd retinas at 3, 5, 9, and 12 months. (B–D) Graphs showing the effect of frequency on response amplitude; for all light-responsive units (3 months, *n* = 769; 5 months, *n* = 605; 9 months, *n* = 614; 12 months, *n* = 694) (B), ON-responsive units only (C), or OFF-responsive units only (D). (E) Heatmaps showing normalized response amplitudes for single units, following modulation of temporal flicker (3 months, *n* = 769; 5 months, *n* = 605; 9 months, *n* = 614; 12 months, *n* = 694). (F) Graph showing mean peak temporal frequency across intensities (3 months, *n* = 547–818; 5 months, *n* = 540–605; 9 months, *n* = 508–616; 12 months, *n* = 535–746). (G) Peak temporal frequency values from single units at 16.9 log photons/cm^2^/s (3 months, *n* = 769; 5 months, *n* = 605; 9 months, *n* = 614; 12 months, *n* = 694). Graphs show mean ± SEM.

Robust responses to the temporal frequency component of the chirp stimulus were observed in ReaChR grm6 rd/rd retinas at all ages, with both ON- and OFF-type units showing modulations in firing across the range of frequencies tested and successfully encoding changing frequency (data shown for 16.9 log photons/cm^2^/s) (Figure 5A; see also Figure S2). Mean modulation amplitude showed a similar relationship with temporal frequency across ages for either the whole population of responsive units (Figure 5B) or when ON and OFF units were analyzed separately (Figures 5C and 5D). At the single-unit level, there was diversity in preferred temporal frequency at all ages, with some units showing either low pass (maximal response to the lowest frequency) or band-pass tuning (maximal responses to an intermediate frequency) (Figure 5E). Analysis of individual frequency response curves revealed a subtle change in preferred temporal frequency with age and across stimulus irradiance (Figure 5F). Thus, there was a significant reduction in preferred temporal frequency with increasing irradiance (two-way ANOVA, F(3, 9,978) = 60.38, *p* < 0.0001) and at 12 months (two-way ANOVA, F(3, 9,978) = 19.40, *p* < 0.0001) (Tukey’s post hoc test, 3 vs. 12 months, *p* ≤ 0.0001; 5 vs. 12 months, *p* ≤ 0.0001; 9 vs. 12 months, *p* ≤ 0.0001) (Figure 5F). An interaction between age and irradiance was also evident (two-way ANOVA, F(3, 9,978) = 3.291, *p* = 0.0005) driven by changes in 12-month responses to the highest irradiance (Figure 5F). When measured at 16.9 log photons/cm^2^/s, the mean preferred frequency of individual responsive units was reduced at 12 months compared to earlier ages (one-way ANOVA, F(3, 2,678) = 13.55, *p* < 0.0001) (Tukey’s post hoc test, 3 vs. 12 months, *p* ≤ 0.0001; 5 vs. 12 months, *p* = 0.0025; 9 vs. 12 months, *p* = 0.0008) (Figure 5F) and showed a significant linear trend, indicating a progressive reduction in peak temporal frequency over time (*R*^*2*^ = 0.0122, slope = −0.0016, F(1, 2,680) = 33.11, *p* ≤ 0.0001) (peak temporal frequency at 3 months = 1.77 ± 0.058 Hz [*n* = 769], 5 months = 1.55 ± 0.061 Hz [*n* = 605], 9 months = 1.58 ± 0.065 Hz [*n* = 614], 12 months = 1.24 ± 0.059 [*n* = 694]).

### Contrast sensitivity and sensitivity normalization

**Figure 6:**
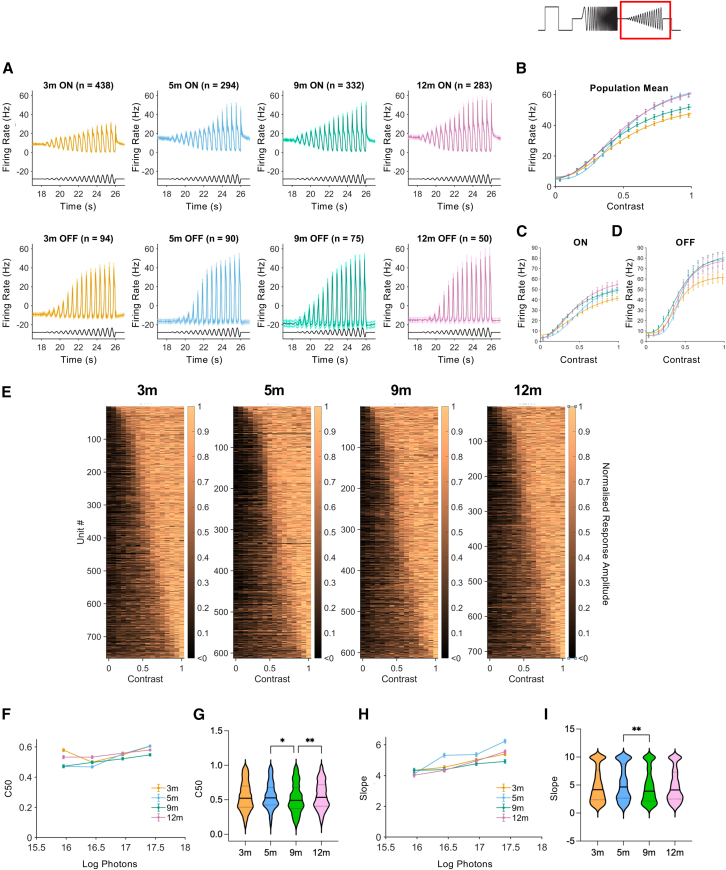
Contrast sensitivity in ReaChR grm6 rd/rd retinas (A) Mean PSTH traces showing responses to modulations of contrast from ON-responsive units (top) and OFF-responsive units (lower panels) of ReaChR grm6 rd/rd retinas at 3, 5, 9, and 12 months. (B–D) Graphs showing the effect of contrast modulations on response amplitude; for all light-responsive units (3 months, *n* = 765; 5 months, *n* = 612; 9 months, *n* = 613; 12 months, *n* = 719) (B), ON-responsive units only (C), or OFF responsive units only (D). (E) Heatmaps showing normalized response amplitudes for single units, following modulations of contrast (3 months, *n* = 765; 5 months, *n* = 612; 9 months, *n* = 613; 12 months, *n* = 719). (F) Contrast values eliciting 50% response amplitude (C50) across intensities (3 months, *n* = 557–848; 5 months, *n* = 486–650; 9 months, *n* = 542–636; 12m *n* = 513–782). (G) C50 values calculated for single units at 16.9 log photons/cm^2^/s (3 months, *n* = 765; 5 months, *n* = 612; 9 months, *n* = 613; 12 months, *n* = 719). (H) Graph showing mean contrast slope values across intensities (3 months, *n* = 557–848; 5 months, *n* = 486–650; 9 months, *n* = 542–636; 12 months, *n* = 513–782). (I) Contrast slope values calculated for single units at 16.9 log photons/cm^2^/s (3 months, *n* = 765; 5 months, *n* = 612; 9 months, *n* = 613; 12 months, *n* = 719). Graphs show mean ± SEM.

Robust responses to elements of the contrast chirp were observed at all ages, with firing patterns (peristimulus time histograms [PSTHs]) of both ON and OFF neurons showing modulations associated with the stimulus (data shown for 16.9 log photons/cm^2^/s) (Figure 6A). Plots of mean contrast response profiles for all units (Figure 6B) or ON and OFF units separately (Figures 6C and 6D) displayed the expected positive relationship between contrast and response amplitude at all ages. These averages encompassed unit-by-unit variation in contrast sensitivity in all age groups (Figure 6E). To determine whether there were age-dependent differences in contrast coding, sigmoidal contrast response functions were fitted at single-unit resolution and across the range of stimulus irradiances to determine the contrast that produced 50% response (C50) and slope. This revealed small but significant effects of irradiance, age, and an age-by-irradiance interaction on C50 values (two-way ANOVA, F(3, 10,078) = 85.33, *p* ≤ 0.0001; F(3, 10,078) = 28.89, *p* < 0.0001; and F(9, 10,078) = 10.94, *p* < 0.0001, respectively) (Figure 6F). While modest increases in C50 as a function of irradiance are detected at 12 months (and to a lesser extent, 3 months), (Tukey’s post hoc test, 3 vs. 5 months, *p* = 0.0001; 3 vs. 9 months, *p* ≤ 0.0001; 5 vs. 12 months, *p* ≤ 0.0001; 9 vs. 12 months, *p* ≤ 0.0001), overall differences in mean C50 between ages at any irradiance are modest. Analysis of the data collected at saturating irradiances (16.9 log photons/cm^2^/s) showed a similar distribution of single-unit C50 values between ages, with only a small reduction in mean C50 values observed at 9 months compared to 5 and 12 months (one-way ANOVA, F(3, 2,705) = 4.249, *p* = 0.0053) (Tukey’s post hoc test, 5 vs. 9 months, *p* = 0.0303; 9 vs. 12 months, *p* = 0.0045), but no consistent change in C50 values with increasing age (*p* = 0.987) (Figure 6G).

Contrast response function slopes also show statistically significant variation according to irradiance (two-way ANOVA, F(3, 10,078) = 86.22, *p* < 0.0001), age (F(3, 10,078) = 20.98, *p* < 0.0001), and irradiance by age (F(9, 10,078) = 5.649, *p* < 0.0001). Slopes tend to become steeper at higher irradiance, while differences between ages were not systematic and were driven largely by steeper slopes at 5 months (and to a lesser extent, 3 months) compared to later time points (Tukey’s post hoc test, 3 vs. 5 months, *p* ≤ 0.0001; 3 vs. 9 months, *p* = 0.0441; 5 vs. 9 months, *p* ≤ 0.0001; 5 vs. 12 months, *p* ≤ 0.0001) (Figure 6H). Importantly, the magnitude of such differences is small, suggesting little functional significance, and again, when comparing data only at higher intensities (data shown for 16.9 log photons/cm^2^/s), the slope of contrast-response function shows only a small change between 5 and 9 months (one-way ANOVA, F(3, 2,705) = 4.143, *p* = 0.0061) (Tukey’s post hoc test, 5 vs. 9 months, *p* = 0.0033), with no linear trend detected across ages (*p* = 0.0852) (Figure 6I). Overall, the distribution of C50 and slope values calculated for individual units is broadly similar in ReaChR grm6 rd/rd retinas across ages (Figures 6G and 6I). The small magnitude of any irradiance effect is consistent with evidence that this optogenetic intervention can support contrast coding across changes in background light.^11^

### Response diversity and community detection

**Figure 7:**
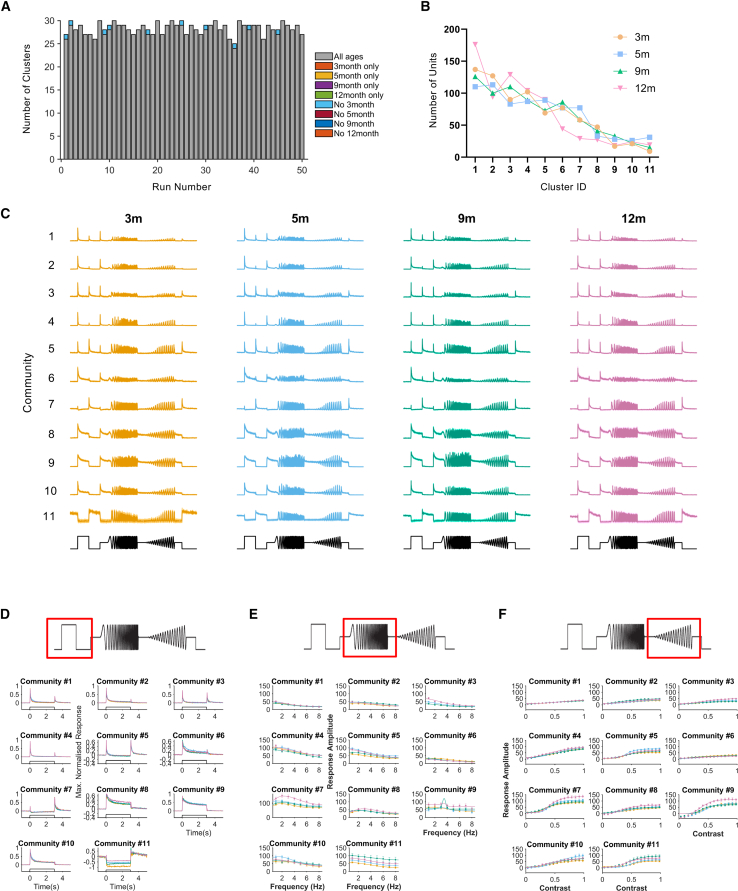
Visual code in ReaChR grm6 rd/rd retinas (A) Graph showing results from 50 separate iterations of probabilistic clustering. Color-coded blocks represent clusters identified in each run not containing units from all ages, while gray shows clusters containing units from all age groups. (B) Graph showing the results of community clustering and the prevalence of units in each cluster in ReaChR grm6 rd/rd retinas at 3, 5, 9, and 12 months of age. (C) Mean PSTH traces for units grouped within each community. (D–F) Comparison of response properties for each community detected in ReaChR grm6 rd/rd retinas at 3, 5, 9, and 12 months of age, showing responses to step stimuli (D), responses to modulation of temporal frequency (E), and increasing contrast (F). Graphs show mean ± SEM.

Our analysis thus far indicates that for the RGC population as a whole, there are only modest differences in visual response properties across the 3–12 month (P30–P270) age range. We finally asked whether differences may be more apparent at a higher-level analysis encompassing the combination of visual response properties. Following previous publications, we performed unsupervised clustering of responses to the full chirp stimulus from RGCs in ReaChR grm6 rd/rd retinas at 16.9 log photons/cm^2^/s pooled across ages using sparse principal-component (PC) analysis and probabilistic clustering, followed by community detection.^11^^,^^60^^,^^65^ Across 50 independent iterations of the clustering, the number of clusters identified varied from 24 to 30 (Figure 7A). Comparing the composition of these clusters across runs revealed that in 40/50 iterations, all clusters contained units from all 4 age groups. In the remaining 10, a single cluster was missing units from the 3-month time point (Figure 7A).

Given the probabilistic nature of the clustering approach, the exact composition of clusters varies from run to run of the analysis. Therefore, to explore the clusters in more detail, we first provided a more stable classification by generating a similarity matrix based on how frequently each unit occurred in the same cluster as every other unit across the 50 repeats of probabilistic clustering. Based on this similarity matrix, community detection was used to group units into functional output channels.^11^ The community detection method identified 11 distinct communities of retinal responses that differed in the magnitude, sign, and persistence of their response to the light step; the phase and amplitude of responses to the chirps; their temporal frequency tuning; and contrast sensitivity (Figures 7B–7F). Heatmaps showing responses of individual units within each community are shown in Figure S4A. Each community contained units from each age group, and notably in approximately equal proportions (Figure 7B). Statistical analysis revealed a small change in the distribution of units across communities at 12 months (chi-squared, 106.7, 30, *p* < 0.0001, although no values are beyond 2× SD of group means), with an increase in the proportion of units in community 1 (and to a lesser extent, community 3) and a small decrease in the proportion of units in communities 6 and 7 (Figure 7B). Comparison of mean PSTH traces shows that the properties of responses in each community are highly comparable in ReaChR grm6 rd/rd retinas at 3, 5, 9, and 12 months (Figures 7C and S4A), with detailed analysis showing highly comparable responses to the light step (Figure 7D), sinusoidal modulations of temporal frequency (Figure 7E), and contrast (Figure 7F) for units within each community across age groups.

## Discussion

To date, optogenetic interventions have for obvious reasons been considered as primarily suitable for patients with end-stage retina degeneration, often long after a complete loss of vision or even light perception, and at a stage in disease where extensive remodeling of remaining retinal circuits has likely occurred (including cell migration, the establishment of aberrant synapses, glial remodeling, and neuronal death).^4^^,^^26^^,^^58^ While studies in animal models at late-stage degeneration^66^^,^^67^ and early results from clinical trials^23^ suggest that optogenetic therapies can restore visual responses in late-stage disease, to our knowledge ours is the first systematic investigation of how distinct phases of retina remodeling might impact restored vision. We find no simple decay in the retina’s capacity to support vision across such remodeling events. Inner retinal thinning and RGC loss at very late stages of degeneration must ultimately impact the restored visual code, but our data suggest that there is a wide window in disease progression, not only for optogenetic interventions but also for other gene- and cell-based therapies^24^^,^^25^ aimed at treating vision loss.

The Pde6b^rd1/rd1^ (rd1) mouse is the best-characterized animal model of retinal degeneration, and our anatomical data are consistent with its well-defined pattern of progressive degeneration.^54^^,^^55^ Thus, the ONL was largely absent at our earliest time point for physiological assessment (3 months), consistent with the predicted completion of phase 1 (loss of rods) and progression of phase 2 (cone degeneration) remodeling at this age. Cone loss is almost entirely complete by 5 months,^54^^,^^55^ with phase 3 remodeling (disruption of inner retinal organization and cell death) progressing from ≅6 months.^26^ Consistent with those data, we find that while gross anatomy of the remaining retina layers appears well preserved in our ReaChR grm6 rd/rd at 5 months, there is increasing dysmorphia across 9 and 12 months. This extends beyond disruption to the layered structure of the inner retina to encompass increases in GFAP labeling, RPE infiltration, formation of hypertrophic Müller cell columns and cell migration.

Optogenetic visual response properties are remarkably stable across these anatomical changes to the inner retina. Indeed, the most impactful changes we observed are the increases in response amplitude, sensitivity, and reproducibility (QI) above 3 months. This finding is consistent with the view that earlier stages of retina remodeling (and specifically the formation of new synaptic contacts between bipolar cells and remaining photoreceptors) may act to preserve function in the face of photoreceptor loss,^68^^,^^69^^,^^70^^,^^71^ perhaps by boosting inner retinal responsivity.^72^ In the untreated retina, this could come at the cost of detrimental hyperexcitability,^73^ and indeed we see increases in baseline activity (under darkness) across the 3- to 5-month time points, but our data indicate that impacts on restored vision are more likely beneficial.

Between 5 and 12 months, changes in the quality of restored vision were minor, suggesting that the therapeutic potential of optogenetics is retained across early to mid-stages of remodeling. There are two important caveats to this conclusion. First, we have applied a restricted range of stimuli, and there may be unprobed aspects of vision that are very sensitive to retinal remodeling. Second, the progression of remodeling is uneven across the retina, and we may have identified more substantial deficits if we had been able to selectively target regions with more advanced degeneration. Nevertheless, our data do confirm that important visual characteristics are retained across at least the major portion of the retina well into phase 3 remodeling. Thus, the ReaChR grm6 rd/rd retina does not lose its ability to encode visual contrast across a range of background intensities; track temporal modulations across a range of frequencies; resolve spatially structured stimuli; or produce a diversity of visual response properties across the ganglion cell population indicative of a rich visual code. Moreover, visual response features known to rely on inner retinal information processing (translation of ON bipolar cell depolarization to both ON and OFF responses among RGCs^74^^,^^75^ and the appearance of DS) are retained even at the latest age tested.^76^^,^^77^^,^^78^ This is not to say that optogenetic vision across this age range is equivalent to that of WTs. Given the choice of optogenetic actuator used in this study, sensitivity is of course substantially reduced,^11^^,^^13^^,^^52^ and RF sizes are smaller than those of intact mouse retina. However, we have recently shown that the ReaChR-driven visual code in this model has many consistencies with that of visually intact mice at 5 months,^11^ and the current dataset confirms that this will be true up to at least 12 months of age.

Our data moreover contribute to the debate concerning the most appropriate cell types to target for optogenetic interventions. Introducing optogenetic activators as early as possible in the visual pathway (in this case in ON bipolars directly post-synaptic to photoreceptors) has the theoretical advantage of allowing restored vision to benefit from the information processing capacity of the inner retina. A theoretical counterargument is that circuit remodeling could produce aberrant processing, making intervention early in the pathway more sensitive to progressive degeneration than strategies targeting RGCs directly. Our findings argue against the latter concern over much of the remodeling process (see also Rodgers et al.^48^).

A growing body of evidence from *in vivo* studies, primarily using spectral-domain OCT, demonstrates that inner retinal remodeling also occurs across human retinitis pigmentosa (RP) of various genetic backgrounds.^79^^,^^80^^,^^81^^,^^82^ Interestingly, this also includes X-linked RP GTPase regulator (RPGR)-associated RP, for which several gene therapy trials have demonstrated improvement in retinal function (for review, see McClements et al.^24^). These findings indicate that also in human disease, such remodeling events do not generally obstruct retinal function. Although encouraging, it is worth noting that OCT imaging lacks the resolution to reveal the cellular basis of remodeling in human disease, yet it is clear from histopathological studies that there is a high degree of similarity in remodeling processes observed between humans and mice.^29^^,^^32^^,^^83^ Moreover, disease progression is apparently highly variable in human RP, depending on the causative gene and the particular mutation. The rd1 mouse, where disease is due to large insertion into the PDE6B gene, causing loss of function, exhibits a particularly rapid disease time course. The degree of inner retinal remodeling present in this model thereby likely exceeds the degree of remodeling that can be expected in many cases of human RP. In this regard, it is encouraging that even the extreme remodeling in the rd1 mouse does not impede optogenetic restoration of visual processing. Thus, the observations made here are likely transferable to the study and treatment of human retina disease with diverse genetic backgrounds.

In summary, we provide evidence that the quality of restored visual code produced by optogenetic therapies is remarkably stable across progressive inner retinal degeneration. Our data indicate that the ability of the retina to transmit and process visual information is retained at stages of degeneration characterized by marked inner retinal cell death and neuroanatomical remodeling. These findings support optimism, not just for optogenetic vision restoration but also for the span of regenerative medicine approaches to restore circuit function following neurodegeneration.

## Materials and methods

This study incorporates electrophysiology data from ReaChR grm6 rd/rd mice at 5 months of age and visually intact C57Bl/6 and C3H mice at 5 months previously published in Rodgers et al.^11^ All data from ReaChR grm6 rd/rd mice at 3, 9, and 12 months of age are newly generated for this study and have not been published previously. Previous data were analyzed alongside new recordings using new criteria to identify light-responsive units.

### Ethics

All experiments were approved by the University of Oxford Animal Welfare and Ethical Review Board and all procedures were conducted in accordance with the UK Home Office Animals (Scientific Procedures) Act 1986 (Project License 30/3371, Investigator License I0AEE55E7) and the ARVO Statement for the Use of Animals in Ophthalmic and Vision Research.

### Generation of ReaChR grm6 rd/rd mice

Here, we use a transgenic mouse model, lox-stop-lox *ReaChR-mCitrine; Grm6*^*cre*^*; Pde6b*^*rd1/rd1*^ (referred to as ReaChR grm6 rd/rd throughout), where the red-activatable variant of channelrhodopsin (ReaChR)^13^^,^^52^ is expressed throughout the entire population of ON bipolar cells of retinal degenerate *Pde6b*^*rd1/rd1*^ (rd1) mice.^11^ This transgenic model allows the functional properties of ON bipolar targeted optogenetic responses to be probed over time without the confounds of variable viral gene delivery. ReaChR grm6 rd/rd mice (Tg(Grm6-icre)1Rlbn; B6.Cg-*Gt(ROSA)26Sor*^*tm2.2Ksvo*^/J; Pde6b^rd1/rd1^) were generated at the University of Oxford by crossing a floxed ReaChR mouse line B6.Cg-Gt(ROSA)26Sortm2.2Ksvo/J mice (also known as Rosa26 CAG-LSL-ReaChR-mCit, Jax lab strain: 026294) with a Grm6-Cre.rd1 mouse strain created and maintained at the University of Oxford. The Grm6-Cre strain was originally created in the lab of Robert Duvoisin and R. Lane Brown at Oregon Health and Science University^84^ (MGI: 4411993). Mice were bred to be homozygous for floxed ReaChR transgene and either Cre null (not expressing ReaChR) or heterozygous for Cre (expressing ReaChR under control of the grm6 promoter). Additionally, mice were bred to be homozygous or heterozygous for Pde6b^rd1^, producing mice with or without retina degeneration (termed ReaChR grm6 rd/rd and ReaChR grm6 rd/+). WT data shown for RF and DS measurements was recorded from WT C57Bl/6 mice (Envigo, *n* = 8 retinas) and supplemented with data from *ReaChR; Grm6*^*WT/WT*^; *Pde6b*^*rd1/WT*^ (*n* = 4 retinas), as previously reported.^11^

### Genotyping

Genotyping of mice for rd1, ReaChR, and Cre genes was performed using ImmomixRed mastermix (Bioline) followed by gel electrophoresis. The primer sequences were as follows: Cre (forward 5′-TCAGCAGGTTGGAGACTTTC-3′, reverse 5′-TTCACAACCTGTCAGACCAC-3′, 800-bp band), *rd1* (forward 5′-TGACAA TTACTCCTTTTCCCTCAGTCT-3′, WT reverse 5′-GTAAACAGCAAGAGGCTTTATTG GGAA-3′, *rd1* reverse 5′-GCATTAATTCTGGGGCGCATG-3′, WT 400-bp band, *rd1* 550-bp band), and *ReaChR* (forward 5′-CTTCCCTCGTGATCTGCAA-3′, WT reverse 5′-CAGGACAACGCCCACACA-3′, ReaChR reverse 5′-GTTATGTAACGCGGAACTCCA-3′, WT 96-bp band, *ReaChR* 140-bp band).

### Animal husbandry

Cohorts of mice aged 3, 5, 9, and 12 months were housed under 12-hour light/dark cycle (<50 lux) at 21°C, with food and water available *ad libitum*. We note that 50 lux is several orders of magnitude below the threshold for ReaChR activation, and it is therefore expected that optogenetic light responses are not activated in these mice prior to *ex vivo* examination of retina function. This largely precludes the possibility that activation of retinal pathways may influence rates of disease progression and retina remodeling prior to functional assessment.

The ReaChR grm6 rd/rd mice used were of mixed sex and aged 3 months (*n* = 8 retina, 5 female, 3 male, mean age 95.8 ± 1.9 days, minimum 89 days, maximum 102 days), 5 months (*n* = 5 retina, 4 female, 1 male, mean age 158.6 ± 4.6 days, minimum 144 days, maximum 169 days), 9 months (*n* = 6 retina, 3 female, 3 male, mean age 281.5 ± 6.0 days, minimum 269 days, maximum 311 days), and 12 months (*n* = 7 retina, 2 female, 5 male, mean age 368 ± 2.2 days, minimum 363 days, maximum 375 days). Typically, for each mouse, one eye was used for MEA electrophysiology and one eye was collected for IHC analysis.

### IHC

Immunostaining of retina sections was performed as described previously.^11^ Visualization of ReaChR.mCitrine was enhanced using a chicken polyclonal anti-GFP antibody that also recognizes mCitrine (1:1,000, AVES lab, GFP-1020). Rod ON bipolar cells were labeled with rabbit anti-PKCα antibody (1:1,000, Abcam, catalog no. ab32376). Cone photoreceptors were labeled with goat anti-UVS cone opsin antibody (1:1,000, Santa Cruz Biotechnology, catalog no. sc-14363) and rabbit anti-MWS cone opsin antibody (1:1,000, Millipore, catalog no. AB5405) diluted 1:1,000. Müller cells were labeled with a rabbit anti-GFAP antibody (1:1,000, Abcam, catalog no. ab7260). Alexa Fluor-labeled anti-rabbit and anti-goat secondary antibodies (Thermo Fisher Scientific) were diluted 1:200. Nuclear counterstaining was performed with 4′,6-diamidino-2-phenylindole (DAPI) (Thermo Fisher Scientific) at 500 ng/mL for 5 min. Images were collected using an inverted LSM 710 laser scanning confocal microscope (Zeiss) and Zen 2009 image acquisition software (Zeiss). Individual channels were collected sequentially. Laser lines for excitation were 405, 488, and 561 nm, with emissions collected between 440 and 480 nm, 505 and 550 nm, and 580 and 625 nm for blue, green, and red fluorescence, respectively. Images were collected using a 40× objective, with images collected every 1 μm in the *z* axis. Global enhancement of brightness and contrast was performed using ZenLite 2011 software (Zeiss).

### MEA recordings of retina explants

Following enucleation, retinas were dissected under dim red light (>610 nm) and transferred to glass-bottomed MEA chambers (Multi Channel Systems), with the ganglion cell side facing down. MEA chambers (containing 252 electrodes, each 30 μm in diameter and spaced 100 μm apart) were placed into the MEA recording device (MEA2100-256 system, Multi Channel Systems) and positioned within the light path of an inverted Olympus IX71 microscope. Retinas were perfused with Ames’ media bubbled with 95% O_2_/5% CO_2_ (pH 7.3) and maintained at 34°C. Recorded signals were collected, amplified, and digitized at 25 kHz using MCS Experimenter Software (Multi Channel Systems). Retinas were perfused for at least 30 min in darkness before commencement of experiments.

### Visual stimuli paradigms

A white light-emitting diode (LED) light source with a daylight spectrum (ThorLabs, SOLIS-3C) and an arbitrary waveform generator (Silgent RSDG2000X, RS components) were used to generate chirp light stimuli,^60^ consisting of 3-s steps from dark to 100% intensity, followed by 2 s of dark, 2 s at 50% intensity, 8 s of temporal chirp (accelerating sinusoidal modulation at 100% contrast from 1 to 8 Hz at 1 Hz/s), 2 s at 50% intensity, 8 s contrast chirp (2 Hz sinusoidal modulation from 3% to 97% contrast), 2s at 50% intensity, and 3 s of dark. Chirp stimuli were presented from lowest to brightest intensity and repeated 10× at each light intensity. Intensity of light stimuli ranged from 14.9 to 17.4 log photons/cm^2^/s and was controlled via motorized filter wheels containing neutral density filters (0–4 log units, ThorLabs).

Sparse spatial noise and moving bar light stimuli were generated as described previously.^11^^,^^41^ Briefly, a narrow-band 565-nm OptoLED light source (Cairn Research) was used in combination with a digital mirror “pattern stimulator” device (Polygon400, Mightex Systems) to create sequences of defined light patterns. The light intensity was 16.2 log photons/cm^2^/s, 565 nm LED, the contrast between bright and dark fields was 1:1,000. RFs were mapped using a sparse binary noise stimulus, applied as a 16 × 16 chessboard pattern with a 48.8-μm-wide pixel width (256 consecutive frames), with a frame duration of 1 s. DS was assessed using a sequential projection of 2-mm-wide moving bars in 4 distinct directions (0°, 90°, 180°, and 270°), at a rate of 500 μm/s (total time 4 s).

### Light measurement

The power of all light stimuli (in μW/cm^2^) were measured at the sample focal plane using an in-line power meter (PM160T, ThorLabs), and units converted to photons/cm^2^/s using an irradiance toolbox^85^ (http://www.eye.ox.ac.uk/team/principal-investigators/stuart-peirson). All devices were automatically controlled and synchronized by a Digidata 1440A digital input/output board (Axon Instruments, Molecular Devices) and WinWCP software (version 5 5.5, J. Dempster, Strathclyde University, Glasgow, UK).

### Spike sorting

Spike sorting of retina MEA data was performed using SpikeSorter software (version 7.77b, Nicholas Swindale, University of British Columbia).^86^ Raw data were filtered using a high-pass 4-pole 500-Hz Butterworth filter. Event detection was based on 4.5× median noise signal, with a window width of 0.24 ms. The results of automatic spike sorting were manually inspected and corrected using SpikeSorter software and then Offline Sorter (Plexon) before being exported to MATLAB (version R2023b) for further analysis.

### Identification of LR units

PSTHs with 25-ms bin size were generated. Units with low spike firing rates (<10% of bins containing spiking activity) and spiking activity in <8 trials were excluded from further analysis. LR units were identified using a shuffle test based on correlation across trials with a significance threshold of *p* < 0.0001.

### Response amplitude and irradiance response curves

The normalized firing rate was calculated by subtracting the average baseline firing during 2 s before onset of the 3-s step stimulus. Response amplitude was defined as the maximum normalized firing rate during the 3-s step or 3 s after step stimulus to capture both ON and OFF responses. All units identified as LR at 16.9 log photons/cm^2^/s were included for analysis. Irradiance response curves were constructed by fitting the Hill slope curve with four best-fit parameters (top, bottom, logEC_50_, and slope) identified using non-linear regression.^11^

### RF mapping

For spatial stimuli, units were identified as light responsive based on response to a 1-s full field flash occurring at the end of the protocols. Units were classified as light responsive if there was a change in spike firing rate of >10 spikes/s and a relative change in number of spikes >20% during flash compared to baseline. RF diameter was calculated using the method described previously,^41^ with minor modifications: for each stimulus frame, PSTHs were calculated for an interval starting 500 ms before the beginning of frame to 500 ms after the end of frame (bin size 250 ms). The absolute peak difference in spike firing rate over each PSTH was then calculated, which enabled the analysis of ON, OFF, ON-OFF, and sustained units. Resulting values were multiplied with a two-dimensional binary matrix representing the stimulus pattern during that particular frame to match responses to individual stimulus locations. Resulting matrices were then collapsed and normalized. RF center positions were detected, and the width of the RF was determined by Gaussian fits. RF center positions were detected on smoothed data to minimize the effect of possible noise. RFs that had their calculated maximum at the edge of the field of view were excluded from analysis.

### DS

The DS index was calculated as described previously.^41^^,^^64^ For each responsive unit, the spike firing rate during the presentation of the moving bar was computed for each of the four directions of movement independently. A DS vector for each responsive unit was computed. The length of the vector was normalized, resulting in the DS index.

### Classification of light step responses

PSTHs were generated with a bin size of 25 ms. We used three different confidence limit (CL) tests: activity during the first 2 s of the chirp step compared with 1s before step onset (transient ON), activity during 0.5 s after step offset compared with last 1 s of step stimulus (OFF), and activity during last 1 s of step stimulus compared with 1 s before step onset (sustained ON). Light-responsive units were then classified into four different categories based on outcome of these CL tests: (1) transient ON units were significant for transient ON test only; (2) sustained ON units were significant for transient and sustained ON tests only; (3) biphasic units show significant activation for transient ON and OFF tests, with no significant response to sustained ON test; and (4) OFF/suppressed ON units had significant suppression for transient and sustained ON tests and had significant responses with OFF test.

### QI

To assess response reproducibility, PSTHs with a 200-ms bin size were used to calculate QI values for all LR units.^11^^,^^60^ Calculations of QI were performed as previously described.^48^ Units with a QI <0.1 were excluded from further analysis.

### Response latency

Latency of response onset to step stimuli was defined as timing of first bin to exceed 95% CL in 1 s after onset of step stimulus. This 95% CL was based on 2 SDs of baseline firing during 1s before onset of light step. Units that did not exceed this threshold, such as OFF units, were excluded from this analysis.

### Response polarity and transience

ON-OFF bias and transience index values were calculated for all LR units using previously described methods.^41^^,^^64^ The ON-OFF bias index was used to assess response polarity and was calculated as the ratio of spike firing during 500 ms after onset (ON firing) and 500 ms after offset (OFF firing) of light step. This produces a scale from −1 (responding only to light OFF) to 0 (responding equally for light ON and OFF) to 1 (responding only to light ON). The transience index was used to test response persistence. A PSTH with 25-ms bin size was normalized to maximum firing rate during a 3-s step. Area under the curve was calculated for 1 s after stimulus onset for ON units (defined as ON-OFF bias index > −0.33) or 1 s after stimulus offset for OFF units (ON-OFF bias index < −0.33). This produces a scale from 0 (highly transient) to 1 (highly sustained with identical response across all bins tested).

### Contrast sensitivity

To assess contrast sensitivity, we used PSTH with 25-ms bin size. The response amplitude to each sinusoidal modulation was calculated as maximum firing − minimum firing rate during each period (0.5 s) of the contrast chirp and normalized to response amplitude for 0.5 s before contrast chirp onset and plotted against Michelson contrast and fit with Naka-Rushton function^87^ using least-squares minimization to identify four best-fit parameters (top, bottom, C50, and slope). C50 was constrained between 0 and 1, and slope was constrained between 0 and 10. Only units with curve fits where *R*^*2*^ > 0.5 and spiking in >10% of bins were used for comparison of contrast sensitivity parameters.

### Analysis of temporal frequency tuning

The mean response amplitude (based on maximum firing − minimum firing during each sinusoidal modulation) was calculated for each temporal frequency (PSTH with 25-ms bin size). These data were fitted with half-Gaussian model five free parameters (low baseline, high baseline, Gaussian spread, peak response amplitude, and peak temporal frequency) using least-squares minimization. Peak temporal frequency was constrained between 1 and 8 Hz. Only units with curve fits where *R*^*2*^ > 0.5, spiking in >10% of bins, and Gaussian spread >0.51 were used for a comparison of temporal frequency tuning parameters.

### Clustering and community detection

Sparse PCs (sPCs) were generated for the following windows of chirp stimulus: step (0.5–4.5 s), temporal chirp (6.5–15.5 s), and contrast chirp (17.5–24.5 s), using the SPaSM toolbox as described by Baden et al. and Caval-Holme et al.^60^^,^^65^ We pooled mean PSTHs (25-ms bins) for all light-responsive units from 3-, 5-, 9-, and 12-month groups and extracted up to 30 features with 5 non-zero time bins. We then discarded those that accounted for <1% of the variance. Response features that met these criteria for each window were then combined to produce a total of 21 features. sPCs from pooled data were then clustered with a mixture of Gaussian models, a probabilistic model using random initialization. The optimum number of clusters is determined based on the lowest Bayesian information criteria, which rewards fit but penalizes complexity, and a Bayes factor <6, used as a threshold for when there was no longer evidence for further splitting.^65^ Probabilistic clustering was repeated 50 times, and a pairwise similarity matrix was generated based on how frequently each unit pair clustered together. Units were then grouped into functional output channels by a community detection algorithm^88^ using the Brain connectivity toolbox.^89^ The distribution of units across communities was compared between genotypes using a shuffle test, as described previously.^11^

### Statistical analysis

Unless otherwise specified, graphs show mean ± SEM. The sample size is given in the figure legends and refers to the number of individual spike-sorted units. The statistical analysis was performed using GraphPad Prism (version 10.3.1). As stated, two-way and one-way ANOVAs were used for comparison between groups, with post hoc testing performed using Tukey’s test with multiple test correction. Significance was determined as *p* < 0.05.

## Data availability

The data reported in this paper will be shared by the lead contact upon request. Any additional information required to reanalyze the data reported in this paper is available from the lead contact upon request. ReaChR grm6 rd transgenic mice are available subject to the completion of material transfer agreements.

## Acknowledgments

This work was funded by MRC grant MR/S026266/1 awarded to M.W.H., R.J.L., S.N.P., and S.H. M.L. was funded by grants from the ProRetina Foundation (Pro-Re/Projekt/Gi-Wh-Li.04.2021) and the German Research Foundation (LI 2846/6–1). We thank Robert Duvoisin for kindly sharing founder Grm6 Cre mice.

## Author contributions

S.H., J.R., R.J.L., and M.W.H. designed the study. S.H. performed all experiments. M.L., S.N.P., and J.R. provided tools for data analysis, which was conducted by J.R., M.L., and S.H. S.H., M.W.H., and R.J.L. wrote the manuscript, which was edited and approved by all authors.

## Declaration of interests

R.J.L. and J.R. are named inventors on patent applications for the use of animal opsins in optogenetic vision restoration. R.J.L. has received investigator-initiated research funding from Kubota Vision and has acted as a consultant for Kubota Vision.

## Supplemental information

Document S1. Figures S1–S4

Document S2. Article plus supplemental information
