# Supplementary material for: Optogenetic vision restoration in the face of secondary and tertiary remodeling in the rd1 mouse retina
Source: Mol Ther. 2025 Aug 5;33(11):5840–59. doi: 10.1016/j.ymthe.2025.07.056 (PMC12628169; doi:10.1016/j.ymthe.2025.07.056)
Supplement: Document S1. Figures S1–S4 [file mmc1.pdf]

YMTHE, Volume *33*

Supplemental Information

Optogenetic vision restoration in the face of secondary and tertiary remodeling in the rd1 mouse retina

Steven Hughes, Jessica Rodgers, Moritz Lindner, Stuart N. Peirson, Robert J. Lucas, and Mark W. Hankins

SUPPLEMENTAL MATERIAL

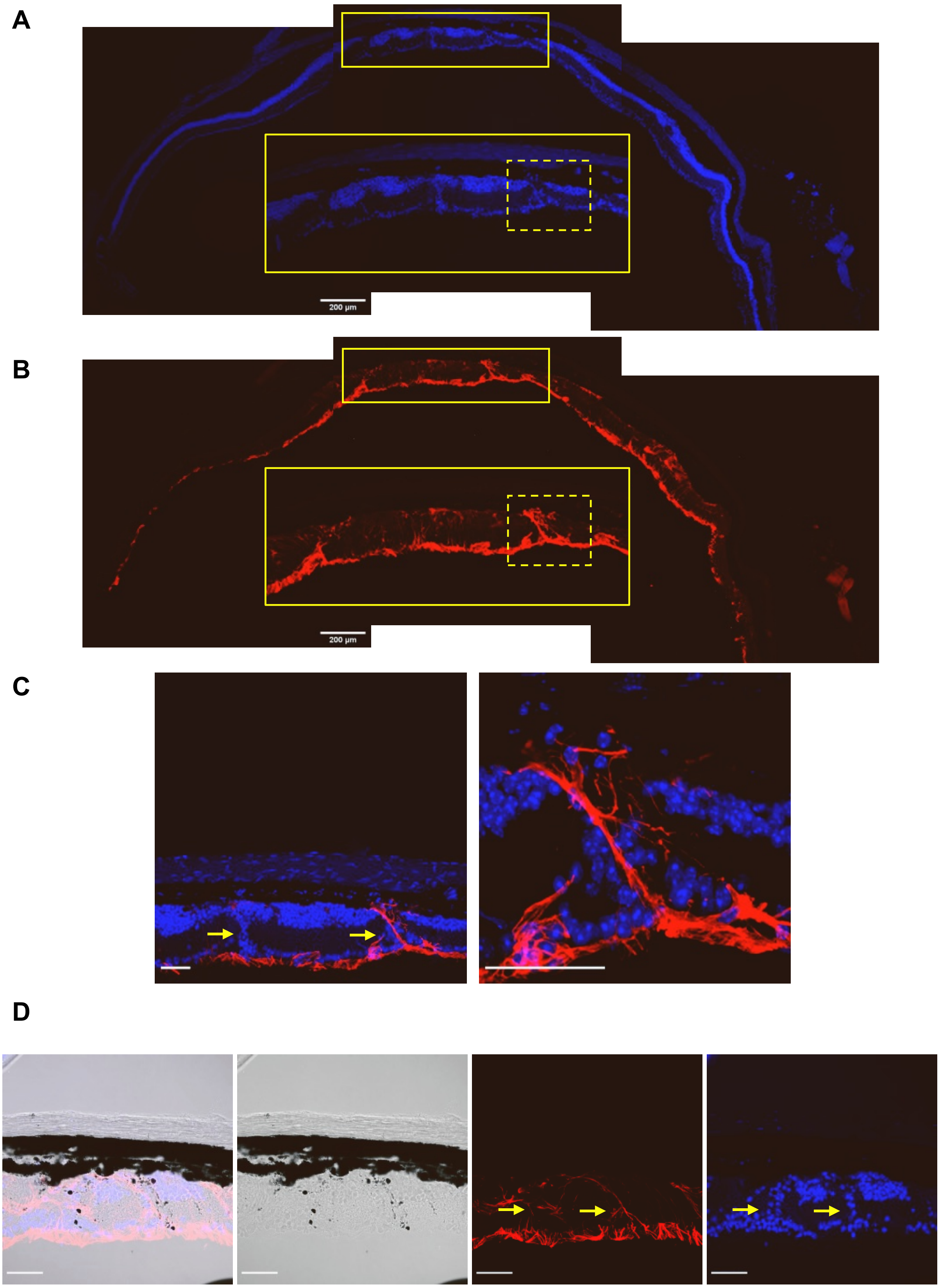

Figure S1. Anatomical changes and retina remodelling in ReaChR grm6 rd/rd retina.

A-B) Assembly of confocal images showing anatomy of retina layers (DAPI nuclei, blue) and GFAP expression (red) across an entire retina section from ReaChR grm6 rd/rd retina at 12 months. Selected regions (solid lines) highlight regions showing significant disruption of retina layers and (dashed lines) the formation of hypertrophic Muller cell columns facilitating mass cell migration. Scale bars show 250µm. **C)** Images showing columns of migrating cell nuclei (DAPI, blue) associated with GFAP (red) reactive Müller cells. Scale bars show 50µm. **D)** Images showing the migration of RPE into the retina (phase contrast), observed in regions of highly remodelled retina characterised by presence of hypertrophic Müller cells (GFAP, red), layer disruption and columns of migrating cell nuclei (DAPI, blue). Scale bars show 50µm.

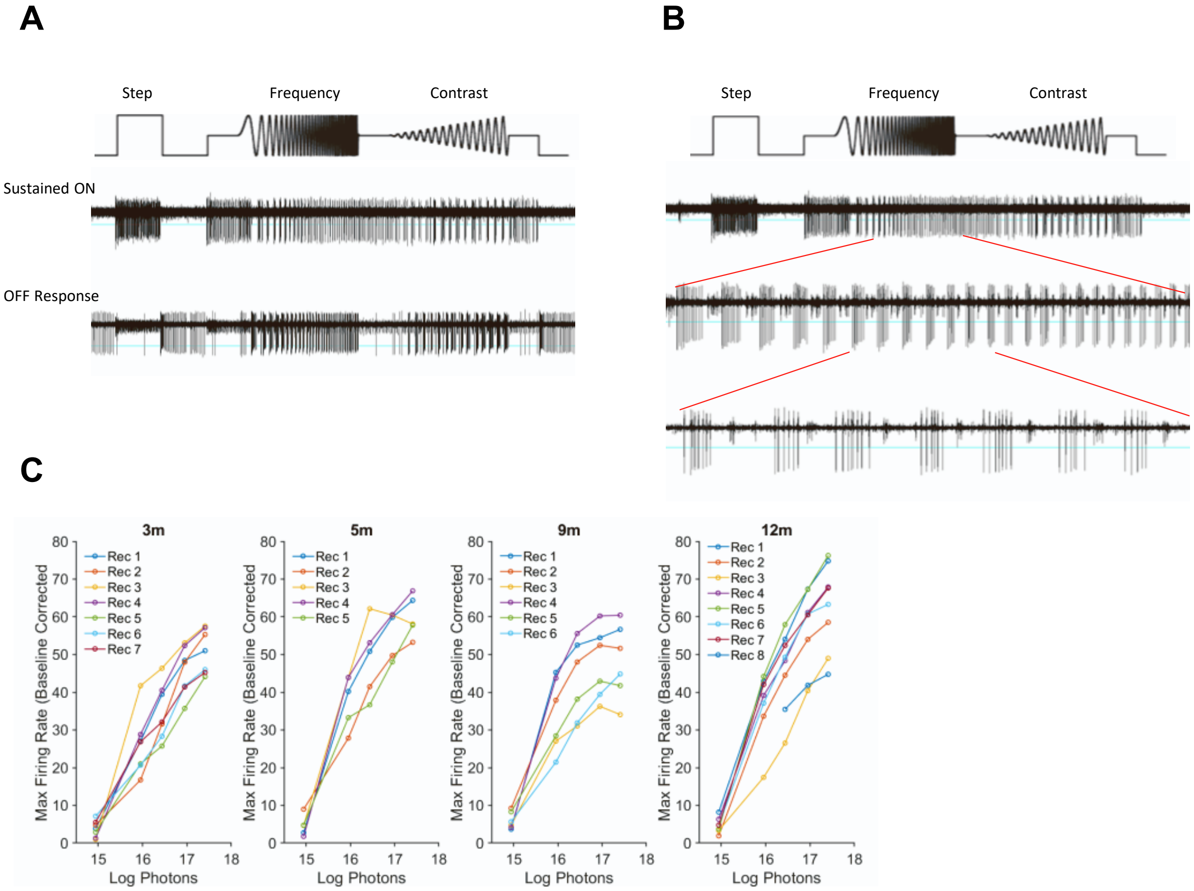

Figure S2. Properties of light responses in ReaChR grm6 rd/rd retina.

A) Examples of raw electrode data showing responses of ON and OFF type units to the entire chirp stimulus, data shown at 16.log photons/cm^2/s. **B)** Example of raw electrode data showing changes in spike firing rate tracking changes in temporal frequency, data shown at 16.log photons/cm^2/s. **C)** Plots of mean response amplitude calculated for each ReaChR grm6 rd/rd retina examined at 3, 5, 9 and 12 months.

A

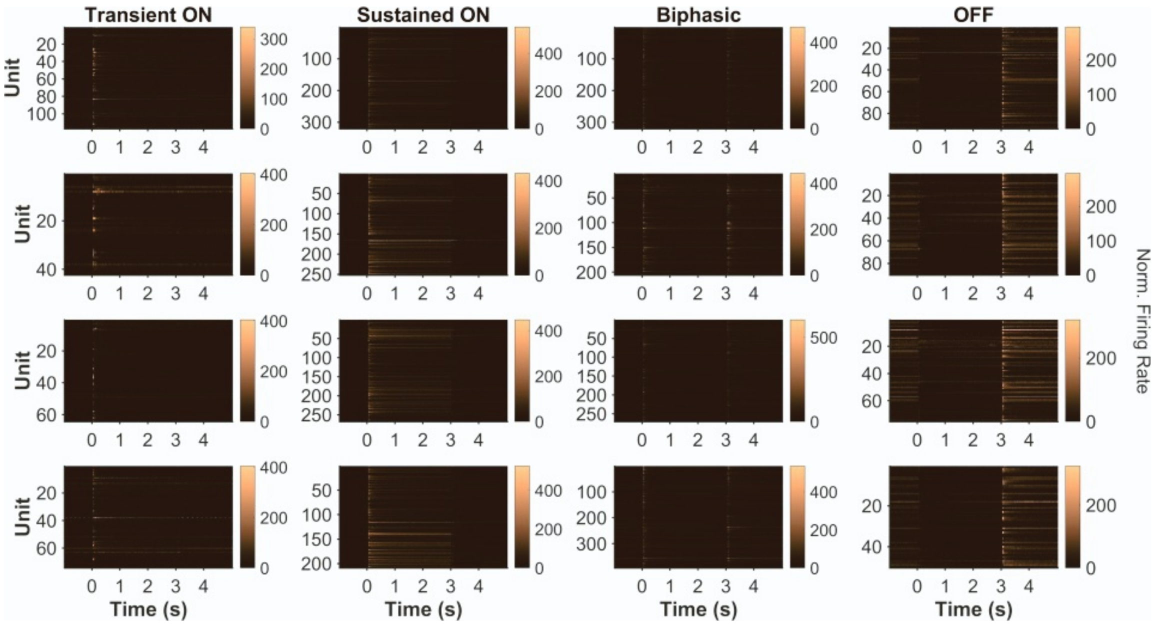

B

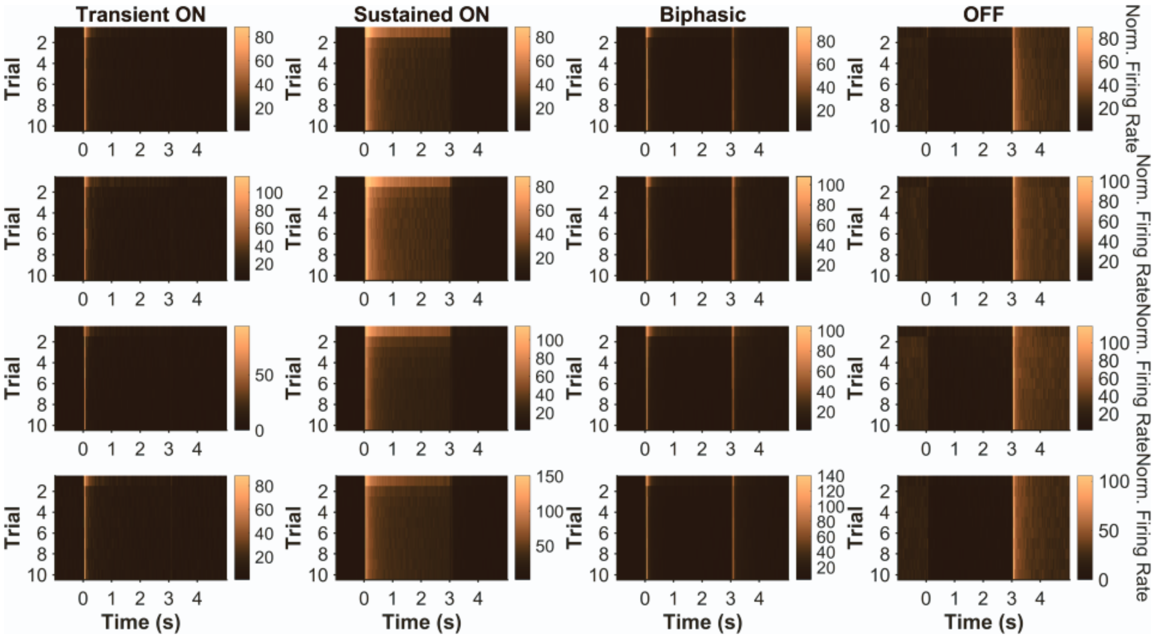

Figure S3. Diversity of step light responses in ReaChR grm6 rd/rd retina.

A) Heat maps showing activity of all individual units classified as Transient ON, Sustained ON, Biphasic and OFF responsive units in ReaChR grm6 rd/rd retina at 3, 5, 9 and 12 months of age. **B)** Heat maps showing the normalised mean activity of all units in each group across the 10 repeats of the light step stimuli. Data shown collected at 16.9 log photons/cm^2/s.

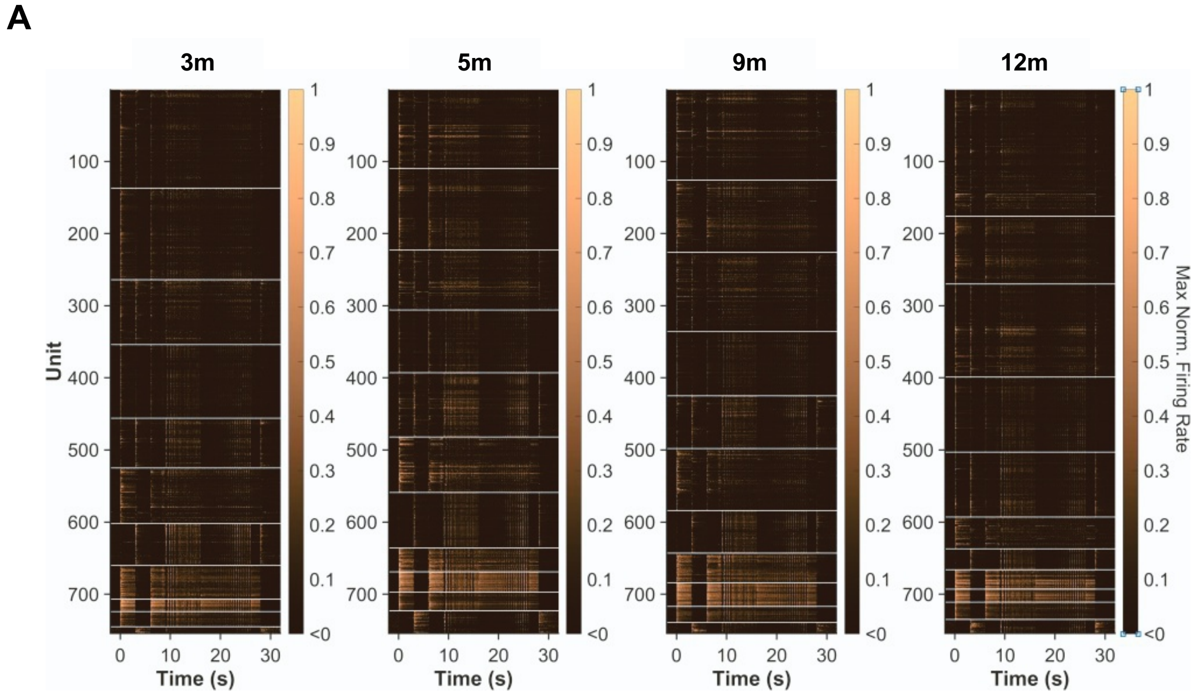

Figure S4 . Functional clustering of light responsive units in ReaChR grm6 rd/rd retina.

A) Heat maps showing the activity (normalised responses) of individual units belonging to each of the 11 functional clusters identified in ReaChR grm6 rd/rd retina at 3, 5, 9 and 12 months of age.
